# Development of antisense-mediated myostatin knockdown for the treatment of insulin resistance

**DOI:** 10.1038/s41598-021-81222-7

**Published:** 2021-01-15

**Authors:** Wouter Eilers, Mark Cleasby, Keith Foster

**Affiliations:** 1 School of Biological Sciences, University of Reading, Reading, UK; 2 Royal Veterinary College, University of London, London, UK

**Keywords:** Metabolic syndrome, Preclinical research, Nucleic-acid therapeutics, Antisense oligonucleotide therapy

## Abstract

Myostatin is a negative regulator of muscle mass and its inhibition represents a promising strategy for the treatment of muscle disorders and type 2 diabetes. However, there is currently no clinically effective myostatin inhibitor, and therefore novel methods are required. We evaluated the use of antisense phosphorodiamidate morpholino oligomers (PMO) to reduce myostatin expression in skeletal muscle and measured their effects on muscle mass and glucose uptake. C57/Bl6 mice received intramuscular or intravenous injections of anti-myostatin PMOs. Repeated intramuscular administration lead to a reduction in myostatin transcript levels (~ 20–40%), and an increase in muscle mass in chow and high-fat diet (HFD)-fed mice, but insulin-stimulated glucose uptake was reduced in PMO-treated muscles of HFD-fed mice. Five weekly intravenous administrations of 100 nmol PMO did not reduce myostatin expression, and therefore had no significant physiological effects. Unexpectedly, exon skipping levels were higher after intramuscular administration of PMO in HFD- than chow-fed mice. These results suggest that a modest PMO-induced reduction in myostatin transcript levels is sufficient to induce an increase in muscle mass, but that a greater degree of inhibition may be required to improve muscle glucose uptake.

## Introduction

Modulation of the expression of specific genes using antisense oligonucleotides (AOs) is a potential therapeutic strategy for diseases of both genetic and non-genetic origins. Hybridisation of AOs to specific complementary sequences on the (pre-)mRNA allows the recruitment of RNAse, which induces degradation of the target transcript or the steric blockade of RNA regulatory proteins, to alter RNA processing. Examples of how this could modify disease phenotypes include the manipulation of specific signalling pathways or the correction of aberrant RNA splicing caused by genetic mutations. In recent years, ten AO-based therapies have received market approval, nine of which are currently marketed. However, most of these therapies target transcripts in the liver, because this is the primary site of AO accumulation after systemic administration^1^. Targeting of other tissues and transcripts has necessitated local delivery; for example, intrathecal administration to target the central nervous system^2^ or intra-orbital administration for retinal disorders^3^. The major exception to this is the use of AOs for Duchenne muscular dystrophy (DMD), the aim of which is to target the dystrophin transcript in skeletal and cardiac muscle cells to restore expression of a truncated version of the dystrophin protein by modifying the pre-mRNA splicing pattern^4^. The development of AO-based therapies able to target genes in a body-wide fashion is of great potential importance for the treatment of non-genetic diseases, because of the relative ease with which an antisense sequence specific to an RNA target of interest can be designed. Skeletal muscle is a good example of a tissue which could be targeted by effective systemic AO-based therapies, given its importance for the maintenance of mobility during aging, the lack of therapies available to counteract muscle wasting conditions, including sarcopenia and cancer cachexia, and its importance for normal metabolism, by virtue of its role as a major store of protein and glucose.

Insulin resistance (IR) is an essential pre-requisite for the development of type 2 diabetes (T2D), a disease which is reaching pandemic status and represents a huge healthcare challenge^5^. IR in skeletal muscle is of particular significance, because this tissue accounts for the majority of post-prandial glucose disposal. IR commonly develops secondary to, or in association with obesity, the prevalence of which is rapidly increasing worldwide. Whereas resistance exercise can ameliorate IR, in addition to counteracting sarcopenia^6^, compliance requires supervised interventions, for which there are insufficient healthcare resources. Thus, effective therapies are required to reverse sarcopenia or to maximise the impact of activity, in order to maintain muscle mass.

Myostatin/growth-differentiation factor-8 (MSTN) is a member of the transforming growth factor (TGF)β superfamily that is synthesised in and secreted from skeletal muscle, where it has inhibitory effects on differentiation and growth, as demonstrated by the pronounced effects of gene-inactivating mutations in multiple species^7–11^. Conversely, greater expression of MSTN is a key feature of cachexia^12–14^ and sarcopenia^15,16^. We and others have demonstrated that postnatal inactivation of MSTN results in hypertrophy, and a consequent increase in muscle mass and strength, along with an increase in insulin sensitivity^17,18^. MSTN also seems to have a selective effect upon adipogenesis^19,20^ and genetic or pharmacological inactivation of MSTN increases lipolysis and fatty acid oxidation in peripheral tissues^21^. Thus, therapeutic MSTN silencing has the potential to ameliorate IR via dual mechanisms: an augmentation of muscle mass and a reduction in adipose mass.

Attempts at inhibiting MSTN action by targeting its receptor (ActRIIB) showed some therapeutic potential^22^. However, recent clinical trials using a soluble ActRIIB decoy in healthy and dystrophic individuals were associated with unacceptable adverse events (http://clinicaltrials.gov/ct2/show/NCT01099761), likely due in part to a lack of specificity, because many other TGFβ family members signal through ActRIIB. Furthermore, while clinical trials using anti-myostatin antibodies or adnectins to inhibit myostatin in a variety of disease contexts have shown small increases in lean body mass^23^, they often fail to demonstrate improvements in muscle function^24^ leading to a halt in their development. Therefore, alternative specific approaches to MSTN inhibition are required. Of these, AO-induced exon skipping, aiming to disrupt the reading frame of the MSTN transcript, is particularly promising. Phosphorodiamidate morpholino oligomers (PMO) have received FDA approval for DMD^25–27^. The PMO chemistry of AO has excellent safety and toxicological profiles and is stable, making it suitable for clinical use. Furthermore, we have previously demonstrated that reducing MSTN gene expression by peptide-conjugated PMO-induced exon skipping induces muscle hypertrophy in mice^28^.

In this study, we aimed to evaluate myostatin exon skipping using antisense PMOs as a potential therapeutic strategy by determining its effects on muscle mass and whole-body and skeletal muscle insulin sensitivity in mice.

## Results

### PMO-induced myostatin exon skipping in skeletal muscle

**Figure 1:**
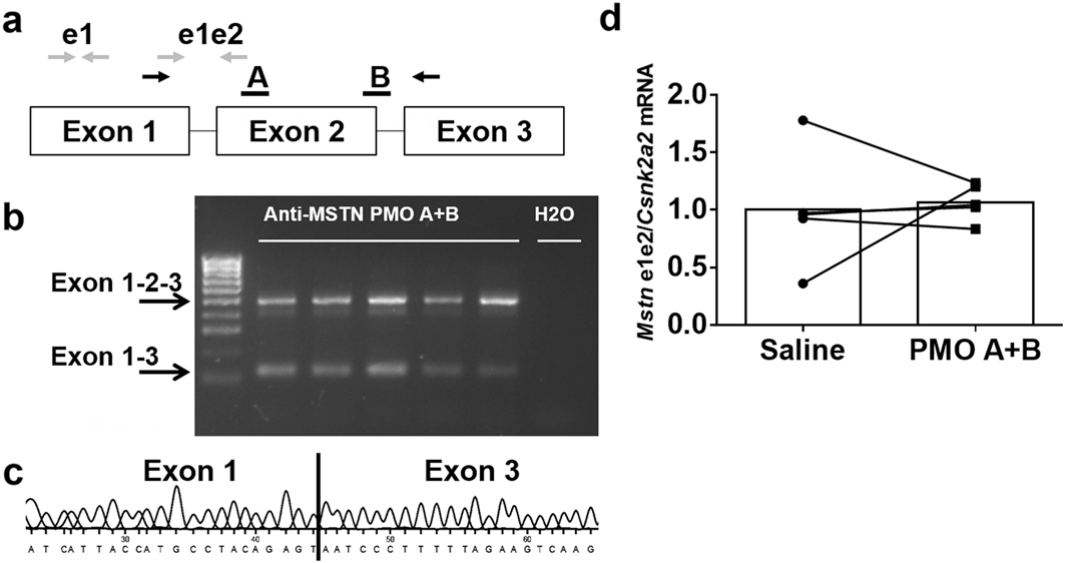
Exon skipping induced by anti-myostatin PMOs. (**a**) Schematic representation of the mouse myostatin transcript, showing exons (boxes) and introns (lines), and indicating the approximate location of the target sequences of the two PMOs used (**a**,**b**, black bars) and the primer pairs used to amplify an exon 1 fragment (grey arrows labelled e1), an exon 1–2 fragment (grey arrows labelled e1e2), or an exon 1–3 fragment (black arrows) by RT-PCR. (**b**) C57Bl/6 mice (n = 5 per PMO) received an intramuscular injection of 5 nmol of anti-myostatin and 1 week later exon skipping was evaluated by RT-PCR. The unskipped transcript amplifies as a 500 bp product and the skipped transcript as a 126 bp product. “H_2_O” indicates the non-template control. Black arrows indicate the expected position of the unskipped (exon 1–2–3) and skipped (exon 1–3) transcript amplicons. (**c**) Result of Sanger sequencing of the 126-bp PCR product obtained from PMO-treated muscle. The sequence corresponding to the 3′ end of exon 1 and the 5′ end of exon 3 is indicated. (**d**) Unskipped myostatin transcript expression following a single intramuscular administration of PMO. Data are shown as individual data points, with data from the same animals connected by lines.

We evaluated a number of PMOs complementary to exon 2 or overlapping the splice donor site of intron 2 of the mouse myostatin transcript, on the basis of the location of binding sites for serine/arginine-rich splicing factors predicted using ESEfinder v3.0^29,30^ by administering a single local injection into the cranial compartment of the lower leg, which includes the TA and EDL muscles, followed by RT-PCR analysis of myostatin exon 2 skipping 1 week post-injection. We found that a mixture of two PMO 30-mers (Fig. 1a) resulted in the highest level of exon skipping in the TA muscle when compared to single PMOs at an equimolar total PMO concentration (data not shown). Exon skipping was verified by gel electrophoresis and DNA sequencing (Fig. 1b,c). However, a single injection was insufficient to significantly reduce the expression of full-length myostatin transcript in the TA muscle (Fig. 1d).

### Repeated intramuscular administration of anti-myostatin PMO increases muscle mass

**Figure 2:**
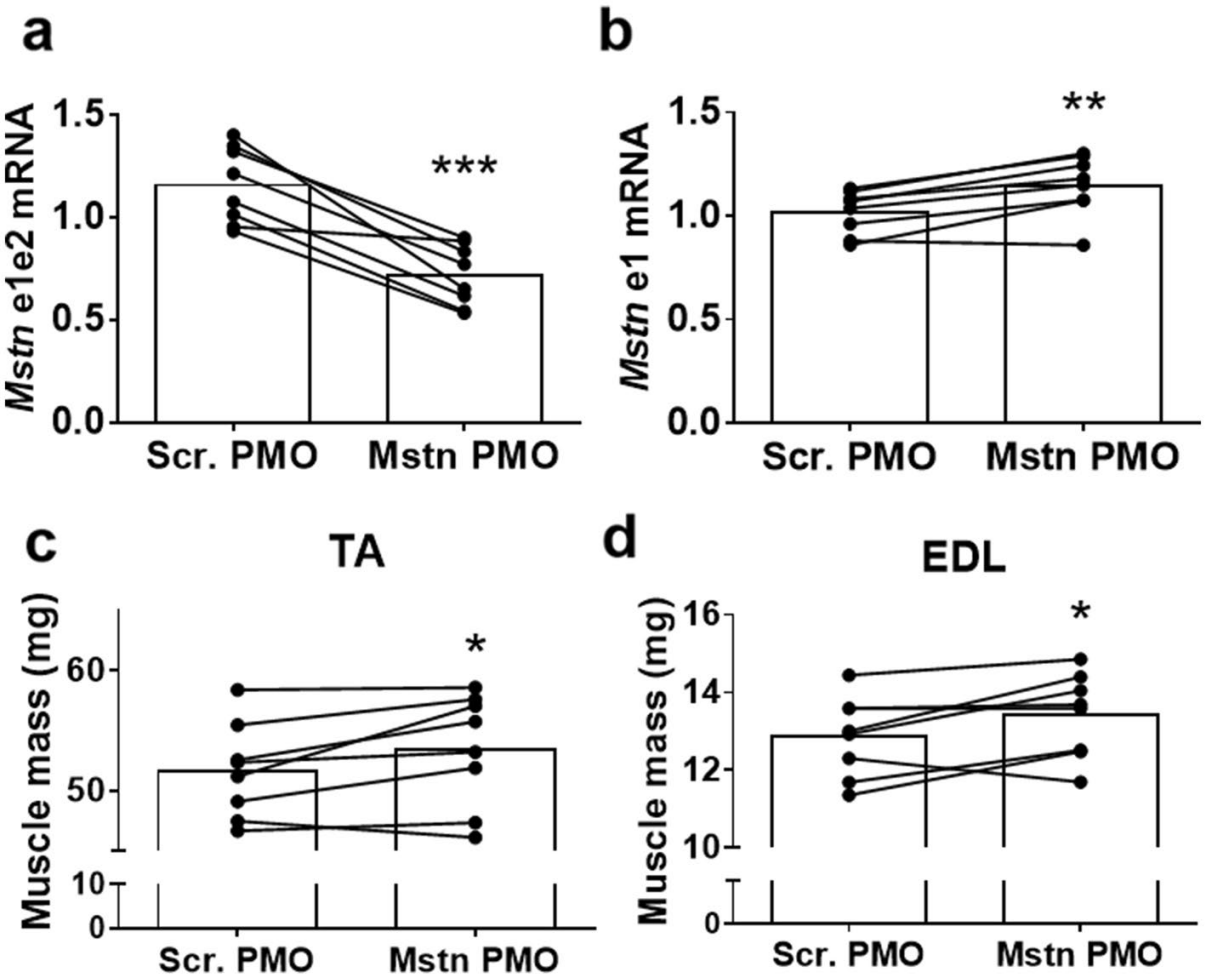
Four weeks of weekly PMO injections reduce full-length myostatin transcript and increase muscle mass. (**a**) Relative full-length myostatin transcript (e1e2 fragment) expression in muscles treated with anti-myostatin (Mstn) or scrambled (Scr.) PMO. (**b**) Relative total myostatin transcript (e1 fragment) expression in muscles treated with anti-myostatin (Mstn) or scrambled (Scr.) PMO. C&D: TA (**c**) and EDL (**d**) muscle masses after treatment with Mstn or Scr. PMO. Data are shown as individual data points, with data from the same animals connected by lines. Bars indicate the mean value for each group. *p < 0.05, **p < 0.01, ***p < 0.001 vs. Scr. PMO. N = 8 per group.

To determine whether repeated PMO administration reduces full-length myostatin transcript expression, we performed four weekly intramuscular TA injections of anti-myostatin or scrambled PMOs to 5-month-old chow-fed C57Bl/6 mice (‘Experiment 1’ in the Methods section). Full-length myostatin transcript expression in the TA muscles was 38% lower (*p* < 0.001) in the myostatin PMO-treated muscles (Fig. 2a), although there was a 13% increase (*p* < 0.01) in the total myostatin transcript expression (Fig. 2b). The TA and EDL masses in the myostatin PMO-treated leg were significantly higher (+ 3.5 and + 4.2%, respectively; *p* < 0.05; Fig. 2c,d). These data demonstrate that the effect of the anti-myostatin PMOs increased during the course of the experiment and that muscle mass can be increased by myostatin exon skipping, even if only a reduction in, rather than a complete elimination of, full-length myostatin transcript was achieved.

### High-fat diet feeding increases the level of PMO-induced exon skipping

**Figure 3:**
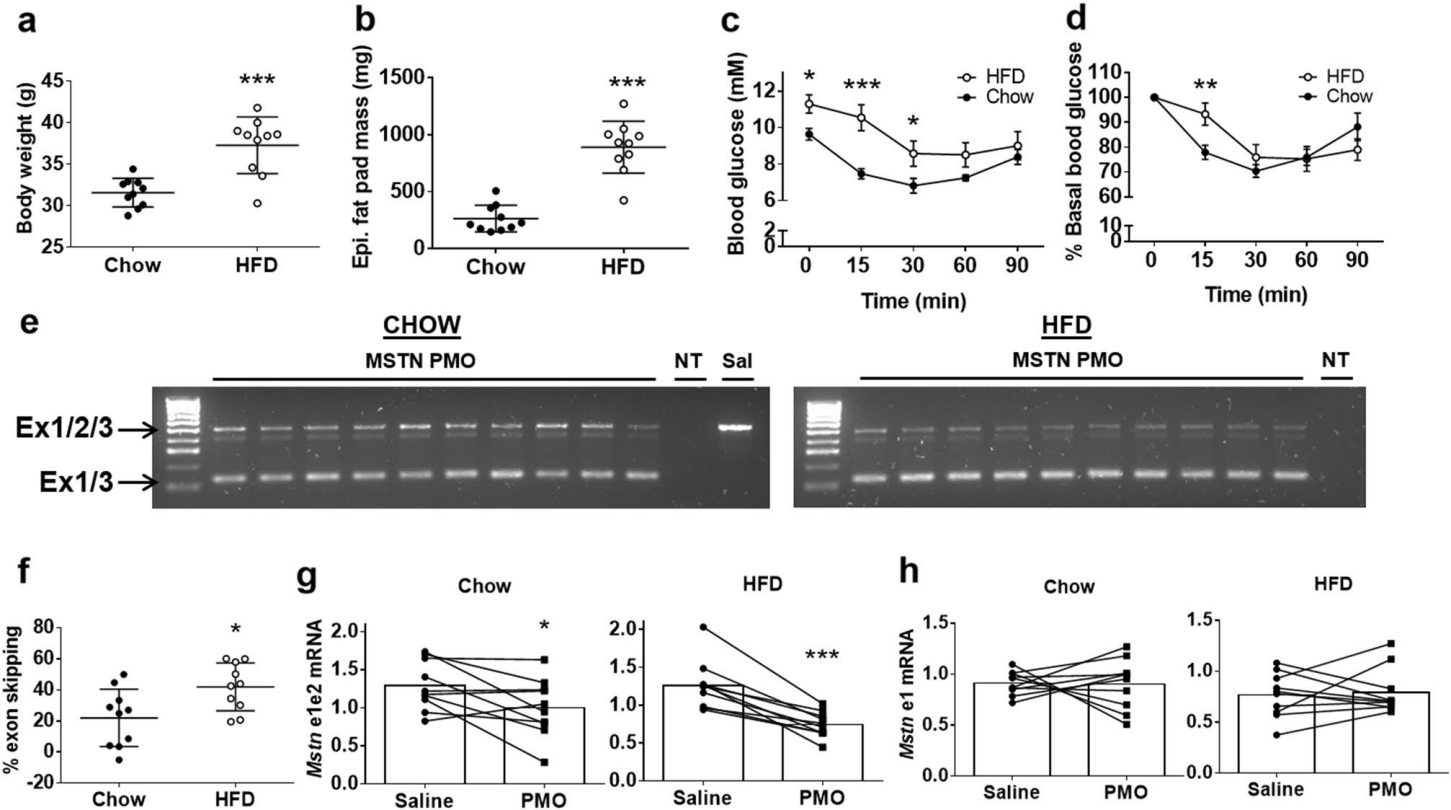
Higher levels of myostatin exon skipping in the muscles of high-fat diet-fed than chow-fed mice after repeated PMO injection. (**a**–**d**) Body mass (**a**); Epididymal fat pad mass (**b**); Absolute blood glucose concentrations during IPITT (**c**); and Relative blood glucose concentrations during IPITT (**d**) of chow- and high fat diet (HFD)-fed mice. (**e**) RT-PCR/agarose gel analysis of myostatin exon skipping. Ex1/2/3: Unskipped myostatin transcript; Ex1/3: Skipped myostatin transcript; NT: non-template control; Sal: Saline-treated muscle. The two gel images shown are unmodified parts of the same original gel image. (**f**) Calculated percentage exon skipping of the myostatin transcript in chow- and HFD-fed mice. (**g**,**h**): Unskipped (**g**) and total (**h**) myostatin transcript expression after repeated PMO administration. Data are shown as individual data points and means ± S.D. (**a**,**b**,**f**), as means ± S.E.M. (**c**,**d**) or as individual data points with bars indicating the mean values and data points from the same animals connected by lines (**g**,**h**). *p < 0.05, **p < 0.01, ***p < 0.001 *vs*. control. N = 10 per group.

Next, we evaluated the effect of repeated intramuscular myostatin PMO administration on exon skipping levels, muscle mass and insulin sensitivity in chow- and HFD-fed mice (‘Experiment 2’ in the Methods section). The HFD-fed mice had higher body mass (+ 18%, *p* < 0.001, Fig. 3a) and epididymal fat pad mass (+ 238%, *p* < 0.001, Fig. 3b) than the chow-fed mice, as well as higher resting blood glucose concentrations (+ 17%, *p* < 0.05, Fig. 3c) and lower insulin sensitivity (Fig. 3d). Exon skipping was induced in all of the PMO-treated muscles in both diet groups (Fig. 3e). Unexpectedly, exon skipping levels in the HFD-fed mice were significantly higher than in the chow-fed mice (HFD: 42%, Chow: 22%, *p* < 0.05, Fig. 3f). This led to a greater reduction in full-length myostatin transcript in the HFD-fed mice (HFD: − 41%, Chow: − 23%, Fig. 3g). However, there was no difference in the expression of full-length myostatin mRNA between saline and PMO-injected muscles (Fig. 3h). Finally, the total myostatin mRNA expression tended to be lower in HFD-fed mice (*p* = 0.06; Fig. 3h).

### Anti-myostatin PMO increases muscle mass but not insulin sensitivity in chow- and HFD-fed mice

**Figure 4:**
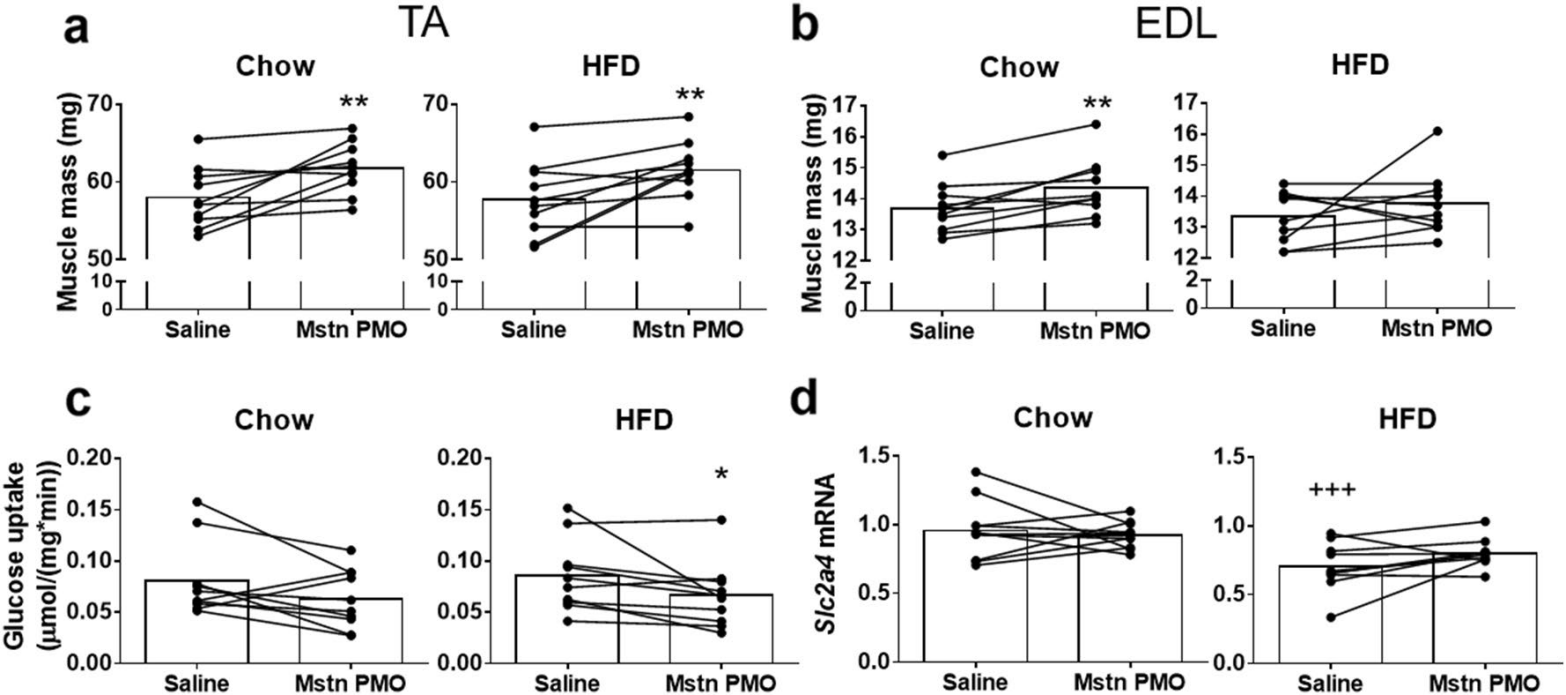
Myostatin exon skipping after repeated PMO injections increases muscle mass, but not insulin sensitivity, in high-fat diet-fed mice. (**a**,**b**) Muscle mass of TA (**a**) and EDL (**b**) muscles from chow- and HFD-fed mice after repeated i/m PMO or saline administration. (**c**) Glucose uptake into TA muscle per unit muscle mass during the IPITT. (**d**) GLUT4 transcript levels in TA muscle. Data are shown as individual data points with bars indicating the mean values and data points from the same animals connected by lines. *p < 0.05, **p < 0.01 *vs*. Saline within same diet group. ^+++^*p* < 0.001 *vs*. saline in the other diet group. N = 10 per group.

TA muscle mass was significantly higher in the myostatin PMO-treated muscles of both chow- and HFD-fed mice (+ 6.6%, *p* < 0.01 and + 6.5%, *p* < 0.01, Fig. 4a). Thus, despite the difference in the reduction in full-length myostatin mRNA achieved between chow- and HFD-fed mice, the increase in mass of the PMO-treated TA muscle was the same in each diet group. However, EDL muscle mass was significantly increased only in the chow-fed mice (+ 4.7%, *p* < 0.05, Fig. 4b). Unexpectedly, we found a significant reduction in glucose uptake into the PMO-treated TA muscles of HFD-fed mice during an IPITT (− 22.6%, *p* < 0.05, Fig. 4c). Transcript levels of *Slc2a4*, which encodes the glucose transporter GLUT4, were lower in saline-injected TA muscles of HFD-fed mice than in the muscles of chow-fed mice (− 27%, *p* < 0.001), but did not differ between PMO-treated and saline control muscles (Fig. 4d).

### Repeated intravenous administration of anti-myostatin PMOs does not reduce myostatin expression sufficiently to increase muscle mass or improve insulin sensitivity

**Figure 5:**
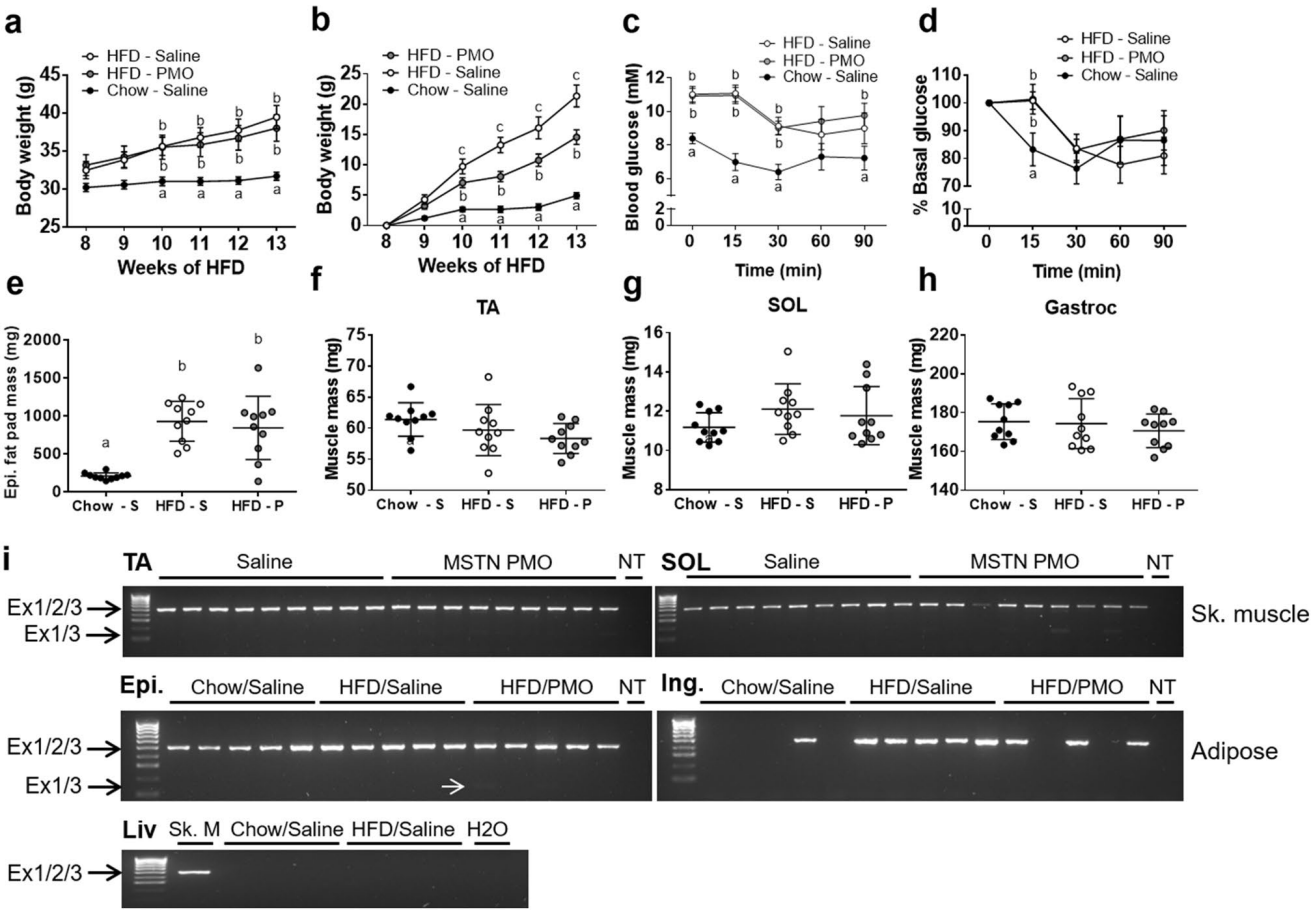
Intravenous administration of unmodified PMOs does not induce sufficient exon skipping to increase muscle mass or decrease fat mass in high-fat diet-fed mice. (**a**,**b**) Body mass (**a**) and percentage increase in body mass (**b**) measured weekly after the start of i/v PMO or saline administration (start: week 8). (**c**,**d**) Absolute (**c**) and relative (**d**) blood glucose concentrations during the IPITT. E–H: Epididymal fat pad (**e**), TA muscle (**f**), SOL muscle (**g**) and Gastrocnemius (**h**) muscle masses in saline-treated chow-fed (Chow-S), saline-treated HFD-fed mice (HFD-S) and PMO-treated HFD-fed mice (HFD-P). (**i**) RT-PCR/agarose gel analysis of myostatin exon skipping in muscle, adipose and liver tissue. Epi: epididymal fat pad. Ing: inguinal fat pad. Liv: Liver; NT: non-template control. Ex1/2/3: Unskipped myostatin transcript; Ex1/3: Skipped myostatin transcript. Small white arrow indicates PCR product from skipped myostatin mRNA. Data are shown as mean ± S.E.M (**a**–**d**), with different letters indicating significant differences between the groups at each time point (*p* < 0.05), or as individual data points and means ± S.D. (E–H). N = 10 per group.

**Figure 6:**
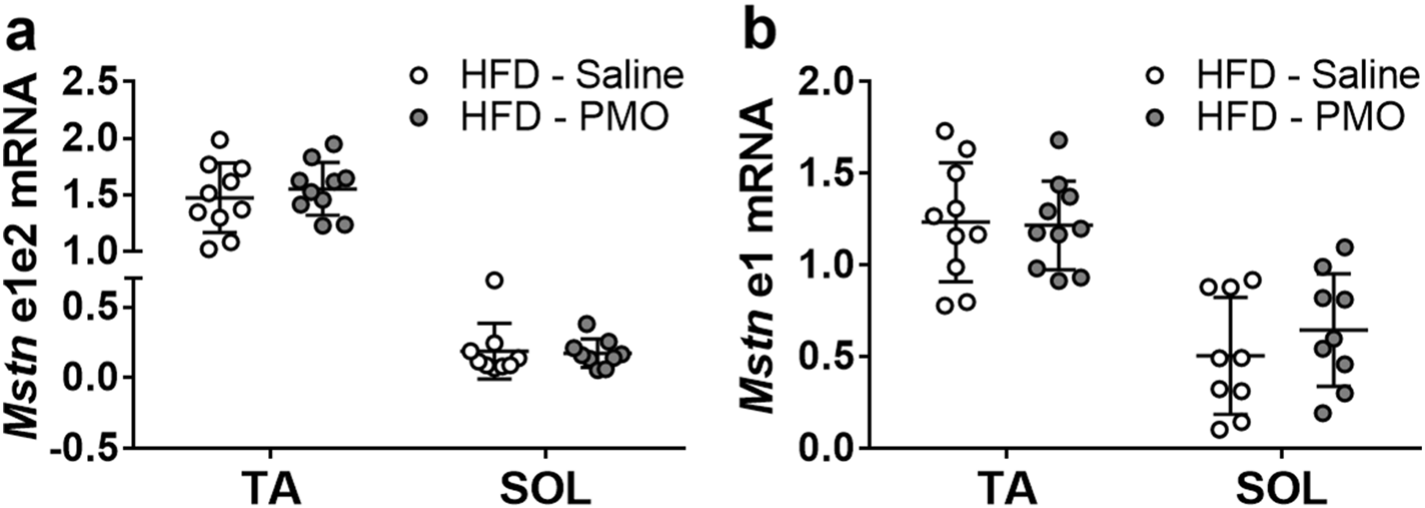
Intravenous administration of unmodified PMOs does not induce sufficient exon skipping to reduce the amount of full-length myostatin transcript. (**a**,**b**) Expression of full-length (**a**) and total (**b**) myostatin transcript in TA and SOL muscle of HFD-fed mice treated with anti-myostatin PMO or saline. Data are shown as individual data points and means ± S.D. N = 10 per group.

To test the feasibility and effects of the systemic delivery of anti-myostatin PMOs in HFD-fed mice, we performed repeated intravenous PMO administrations for 5 weeks (‘Experiment 3′ in the Methods section). The body mass of the HFD-fed mice was higher than that of the chow-fed mice, but was similar in the PMO- and saline-injected HFD-fed mice (Fig. 5a). The relative increase in body mass after the start of the injections was less in the PMO-treated HFD-fed mice than in the saline-treated HFD-fed mice (total increase: 15% vs. 21%, respectively; *p* < 0.05; Fig. 5b). However, there was no effect of PMO administration on blood glucose or insulin sensitivity during an IPITT (Fig. 5c,d). In addition, we found no difference in fat pad mass or the mass of three different muscles between PMO- and saline-treated HFD-fed mice (Fig. 5e–h). When we analysed myostatin exon skipping in the TA and soleus muscles, we detected only very low expression of the skipped myostatin transcript (Fig. 5i) in the muscles of the PMO-treated mice. Consistent with this, we detected no significant differences in unskipped and total myostatin transcript expression in the TA and soleus muscles by real-time PCR (Fig. 6a,b).

Systemic myostatin inhibition could affect other tissues that express myostatin, such as adipose tissue. In addition, it has been suggested that myostatin is expressed in the liver^31^. We therefore analysed myostatin expression and exon skipping in the inguinal and epididymal fat pads and in the liver. Myostatin expression was detected in the epididymal adipose tissue from all the mice, but very low levels of skipped myostatin transcript were present in adipose tissue from only two PMO-treated HFD-fed mice (Fig. 5i). Myostatin transcript was only detected in a subset of the inguinal fat pads and myostatin exon skipping was not detected (Fig. 5i). Finally, myostatin expression was not detected in livers of chow-fed or HFD-fed mice (Fig. 5i) and exon skipping was therefore not examined.

## Discussion

We have evaluated the use of anti-myostatin PMOs to increase muscle mass and improve insulin sensitivity by reducing myostatin expression through the induction of exon skipping of the myostatin transcript. Repeated intramuscular PMO administration lead to a significant reduction in myostatin transcript level and an increase in muscle mass, but no increase in muscle insulin sensitivity in insulin-resistant mice. However, systemic administration of these PMOs did not reduce myostatin expression. Interestingly, a period of high-fat diet feeding increased the level of exon skipping in skeletal muscle.

Local anti-myostatin PMO administration inhibits myostatin signalling by disrupting the expression of myostatin in cells successfully targeted by the PMOs, thereby preventing its autocrine/paracrine action. Thus, there was no inhibition of myostatin expression or release by cells which did not take up PMOs, nor were the effects of myostatin derived from the circulation inhibited. However, our results show that a local reduction in full-length myostatin transcript levels of < 100%, which is therefore presumably reflected in a reduction of the amount of myostatin protein, rather than elimination of its expression, nonetheless resulted in a significant increase in muscle mass (Figs. 2 and 3), suggesting that complete inhibition of myostatin is not required for this effect.

Although anti-myostatin PMOs reduced myostatin expression and increased muscle mass, we did not show an increase in muscle insulin sensitivity in chow-fed mice, and instead found a reduction in insulin sensitivity in HFD-fed mice (Fig. 4). We have previously observed that local myostatin inhibition by adeno-associated virus-mediated overexpression of the inhibitory myostatin propeptide induces an increase in muscle insulin sensitivity in mice on the same HFD feeding protocol as that used in the current study^32^. This was associated with a significantly greater increase in muscle mass (~ 23%) than that achieved following the intramuscular PMO injections (~ 6.5%, Fig. 4), This suggests that although the degree of myostatin inhibition achieved with the local PMO injections was sufficient to increase muscle mass, it was not sufficient to increase muscle insulin sensitivity. Local overexpression of the myostatin propeptide in rats resulted in greater glucose uptake which was associated with a modest (~ 9%) increase in muscle mass^17^. This suggests that large increases in muscle mass are not always necessary to increase muscle glucose uptake. Whether the positive effects of myostatin propeptide overexpression on muscle glucose uptake are due to more efficient inhibition of locally-produced myostatin, the additional effect of blocking myostatin derived from the circulation, or both, is unclear. Myostatin expression and secretion from muscle has been reported to be higher in obese women^33^. However, in our HFD-fed mice myostatin expression in muscle was not higher (Fig. 3h) and therefore, the effects of neither the anti-myostatin PMO, nor the myostatin propeptide, can be ascribed to the counteraction of an obesity-associated increase in myostatin expression.

Unexpectedly, the level of myostatin exon skipping and myostatin knockdown was higher in HFD-fed than chow-fed mice (Fig. 3). It has been previously reported that the incubation of cells with various lipids for several hours increases the activity of AOs with a phosphorothioate backbone by increasing AO leakage from late endosomes^34^. Therefore, it is possible that a similar increase in endosomal leakage of PMOs occurred in the HFD-fed mice. This raises the question of whether increasing dietary lipid content could help increase the efficacy of AOs in clinical studies. Potential corroborating evidence comes from a recent phase I clinical trial for DMD in which a PMO AO targeting exon 53 of the dystrophin gene resulted in substantially higher levels of exon skipping in one patient who had an unusually high body mass, and was described as having a high body fat fraction^35^. However, due to the fact that PMO dose was calculated on the basis of body mass, this patient was administered a larger absolute dose of PMO and had higher serum concentrations of PMO than the other patients in the trial, and therefore it is unclear whether their high body fat content contributed to the level of exon skipping observed.

Despite the apparent increase in PMO efficacy in the HFD-fed mice, there was no difference in the increase in muscle mass achieved in the saline-injected control muscle between chow-fed and HFD-fed mice (Fig. 4). It is possible that, despite the difference in the reduction of myostatin mRNA levels, the reduction in myostatin protein levels was not different between the two groups.

While local intramuscular administration of the PMOs led to a significant reduction in full-length myostatin transcript levels, our protocol of intravenous PMO administration did not (Fig. 6). The PMO backbone is neutrally charged and displays low protein binding compared to antisense oligonucleotide chemistries with ionic backbones^36^. This results in a good safety profile, but also leads to relatively rapid clearance from the circulation by the kidneys which likely compromises its tissue uptake. We previously observed an increase in muscle mass in mice after systemic administration of a octa-guanidine dendrimer-PMO conjugate^28^. However, although the positively charged dendrimer increases exon skipping, it also has significant adverse effects that prevent its translation to the clinic. Systemic PMO treatment of *mdx* mice, a model of Duchenne muscular dystrophy, has been shown to induce transcript modification in skeletal muscles body-wide^37^. However, the mechanism of uptake of the PMOs into muscle cells is likely to be different in these dystrophic mice compared to wild-type mice^38^. Likewise, systemic PMO treatment has been shown to induce transcript modification in the skeletal muscles of a mouse model of spinal muscular atrophy^39^, but these mice are typically treated at the neonatal stage, rather than at adult age. Intraperitoneal administration of PMO in a transgenic mouse model expressing a GFP reporter gene containing an artificial intron resulted in low levels of transcript modification and GFP expression^40^, suggesting that skeletal muscle is not efficiently targeted by systemically administered PMOs. However, it is difficult to compare antisense oligonucleotide efficacy between studies, even when the same oligonucleotide chemistry is used, because of differences in oligonucleotide length, sequence and target transcript. Nonetheless, we detected low levels of exon skipping in skeletal muscle, as well as in epididymal fat deposits, which suggests that increasing the treatment duration, frequency and dose, or improving the AO delivery method, might lead to effective gene silencing. Body mass increased less rapidly in the PMO-treated than in the saline-treated HFD-fed mice (Fig. 5), but is unclear whether this might be due to myostatin knockdown in a tissue not analysed in this study or a non-specific effect based on the PMO chemistry per se. There may have been an effect on myostatin expression in brown fat, which has been suggested to regulate systemic insulin sensitivity^41^, but we did not analyse this tissue in the present study.

We conclude that while it is feasible to increase muscle mass by moderately reducing myostatin transcript levels using PMO AOs, it is unclear what degree of knockdown is required to improve insulin sensitivity. For body-wide effects on muscle mass and insulin sensitivity to occur, larger PMO doses may be required. Future efforts should also focus on ways to improve the efficacy of cellular AO uptake. The observation that a HFD increased exon skipping levels in muscle could inform these efforts. In addition, modifications to the AOs, such as conjugation of peptides or lipids, may enable more efficient cell delivery and intracellular trafficking of AOs, thereby improving their efficacy.

## Methods

### Phosphorodiamidate morpholino oligomer

PMO 30-mers were designed to induce skipping of exon 2 of the murine myostatin transcript by sterically blocking the binding of essential splicing factors. This results in a reading frame shift that prevents translation. A control PMO 30-mer (SCR) that does not have any predicted targets in a BLAST search, or saline, were used as controls. PMOs were synthesised by Genetools (Oregon, USA).

An equimolar mixture of two oligos targeting different regions of the myostatin pre-mRNA was used for these experiments (PMO A: 5′-TCTTGACGGGTCTGAGATATATCCACAGTT-3′; PMO B: 5′-TTTTCAGTTATCACTTACCAGCCCATCTTC-3′; SCR: 5′-GACGGTTCGGCGATAATCCTTCCGCGTCTC-3′).

### Animals

Male C57BL/6 mice (Envigo) were housed in animal facilities at the Royal Veterinary College (RVC) or the University of Reading (UoR) under a 12:12 h day-night cycle, with standard chow or a high fat diet and water available ad libitum. High-fat diet (HFD) was obtained from Research Diets (New Brunswick, USA; #D12451), which contained 45% energy derived from fat, 35% from carbohydrates and 20% from protein. All experimental procedures were approved by the Animal Welfare Ethical Review Body at the RVC or the UoR and conducted under a United Kingdom Home Office licence in compliance with the Animals (Scientific Procedures) Act 1986. All experimental procedures were performed in accordance with relevant guidelines and regulations.

For intramuscular administration of PMO, mice were anaesthetised with isoflurane (4% induction, 2% maintenance) and the cranial aspect of their lower limbs was shaved. PMO was injected into the cranial compartment of the left lower leg via a 29G needle, while the right leg was injected with an equimolar amount of SCR PMO or saline.

For intravenous (i/v) administration of PMOs, conscious mice were restrained and PMO or saline were injected into a lateral tail vein.

### Intraperitoneal insulin and glucose tolerance testing

For intraperitoneal insulin tolerance testing (IPITT), mice were fasted for 3–4 h before the administration of insulin. Insulin was prepared at 100 iu/ml in normal saline and used to resuspend nitrogen-dried 2-[1,2-^3^H(N)]-deoxy-D-glucose (0.37 MBq). Basal blood glucose was measured in tail blood using an Accu-Check Advantage meter (Roche Diagnostics, Burgess Hill, West Sussex, UK). A further 10 µl of blood was collected in a microfuge tube containing 1 iu heparin in saline, mixed and placed on ice. Immediately afterwards, insulin and deoxyglucose tracer was administered intraperitoneally at a dose of 0.75 iu/kg. At 15, 30, 60 and 90 min after insulin administration, blood glucose was measured, as described above. After taking the final blood sample, the mice were euthanised by cervical dislocation and tissues were dissected, weighed and frozen in liquid nitrogen-cooled isopentane. Glucose clearance into TA muscles was determined as described previously^42^.

### In vivo study design for experiments involving repeated PMO administration

Experiment 1: Five-month-old chow-fed male C57BL/6 mice were administered 5 nmol dual anti-myostatin PMO in 50 µl saline weekly for 4 weeks into the anterior compartment of a lower leg, as described above. The contralateral muscles were injected with SCR PMO. One week after the final injection, the mice were euthanised and their tibialis anterior (TA) and extensor digitorum longus (EDL) muscles harvested.

Experiment 2: C57BL/6 mice were switched from regular chow to an HFD at 8 weeks of age, while controls continued to consume a normal chow diet for a further 13 weeks. After 8 weeks of these diets, the mice were administered weekly intramuscular myostatin PMO injections (5 nmol per injection) into one limb and saline injections into the contralateral limb for 4 weeks. IPITTs were performed to assess muscle insulin sensitivity 1 week after the final PMO injection and mice were euthanised and their tissues collected immediately afterwards.

Experiment 3: C57Bl/6 mice were fed an HFD or chow, as described for ‘Experiment 2’, and intravenously injected with 100 nmol PMO or saline once weekly for 5 weeks. One week after the final injection, IPITTs were performed to determine whole-body and muscle insulin sensitivity, and the mice were euthanised and their tissues harvested immediately afterwards.

### RNA analysis

Frozen muscles were homogenised in Tri-reagent (Sigma-Aldrich) and RNA was extracted according to the manufacturer’s instructions. RNA was reverse transcribed using the qScript cDNA synthesis kit (Quanta Biosciences) or the Precision nanoScript2 Reverse Transcription kit (Primerdesign). Transcript levels were quantified in duplicate by real-time PCR using PerfeCta SYBR Green FastMix (Quanta Biosciences). Serial dilutions of a mixture of cDNA from all samples was prepared and targets amplified alongside the samples. The relative amounts of transcript were quantified using a standard curve constructed using the serial dilutions and normalised to *Csnk2a2* mRNA, a reference gene which was selected using geNorm analysis (https://genorm.cmgg.be/). Primer sequences are listed in Table 1.

**Table 1:** Sequences of primers used in RT-PCR.

Transcript	Fw (5′—> 3′)	Rv (5′—> 3′)
*Mstn,* e1	TGTTTATATTTACCTGTTCATGCTGAT	GCCCCTCTTTTTCCACATTTTC
*Mstn,* e1–e2	GAAGATGACGATTATCACGCTAC	CTTTTACTACTTTGTTGTACTGTATTTTAG
*Mstn,* e1–e3	CAGTACGACGTCCAGAGGG	GGCTTCAAAATCGACCGTGA
*Mstn,* e1–e3 nested	GCAGTGATGGCTCTTTGGAA	GTCTCTCCGGGACCTCTTG
*Slc2a4*	ACACTGGTCCTAGCTGTATTCT	CCAGCCACGTTGCATTGTA

Myostatin exon skipping was verified by nested RT-PCR amplification of an exon 1–exon 3 fragment using Quick-Load Taq master mix (New England Biolabs), followed by agarose gel separation of the PCR products, DNA extraction (QIAEX II kit; Qiagen) and sequencing of the PCR product (MWG Eurofins) corresponding to the skipped transcript. Full-length (exon 1–exon2 fragment: e1e2) and total myostatin transcript expression (exon 1 fragment: e1) was determined by real-time PCR, as described above. For intramuscular administration of PMO, exon skipping percentages for each mouse were calculated from the real-time PCR data as follows: % exon skipping = (∆e1 − ∆e1e2)/∆e1*100%, where ∆e1 = e1_PMO_/e1_control_*100% and ∆e1e2 = e1e2_PMO_/e1e2_control_*100%.

### Statistics

Statistical analyses were carried out using SigmaPlot v12.3. Normality and equal variance of the data were tested with the Shapiro–Wilk test and Levene’s mean test, respectively. IPITT blood glucose data (Figs. 3c,d, 5c,d) and transcript levels (Figs. 3g,h and 4d), body mass (Fig. 5a,b), muscle mass (Fig. 4a,b) and muscle glucose uptake data (Fig. 4c) from HFD experiments were analysed by Two-Way Repeated Measures ANOVA. Myostatin transcript levels after i/v PMO administration (Fig. 6a,b) were analysed by Two-Way ANOVA. The Sidak-Holm method was used for *post-hoc* testing, following Two-Way ANOVAs. Tissue mass after i/v PMO administration (Figs. 5e–h) was analysed by One-Way ANOVA, after confirmation of normal distribution and equality of variance. If these assumptions were not met, a Kruskal–Wallis One-Way ANOVA was used. All data from experiments using intramuscular administration of PMO to chow-fed mice (Fig. 2a–d) were analysed by paired *t*-test (two-sided (Fig. 2a,b) or one-sided (Fig. 2c,d)), after confirmation of normal distribution and equality of variance. Body mass (Fig. 3a), fat pad mass (Fig. 3b) and %exon skipping (Fig. 3f) were analysed by unpaired *t*-test after confirmation of normal distribution and equality of variance. Statistical significance was accepted when *p* < 0.05.

## Supplementary information

Supplementary Information.

## Data Availability

The datasets generated during and/or analysed during the current study are available from the corresponding author on reasonable request.
