# Supplementary material for: Development of antisense-mediated myostatin knockdown for the treatment of insulin resistance
Source: Sci Rep. 2021 Jan 15;11:1604. doi: 10.1038/s41598-021-81222-7 (PMC7810755; doi:10.1038/s41598-021-81222-7)
Supplement: Supplementary file 1 — Supplementary Information. [file 41598_2021_81222_MOESM1_ESM.docx]

Supplementary material for

Development of antisense-mediated myostatin knockdown for the treatment of insulin resistance

Wouter Eilers^1*^, Mark Cleasby^2^ & Keith Foster^1^

^1^School of Biological Sciences, University of Reading, Reading, United Kingdom

^2^Royal Veterinary College, University of London, London, United Kingdom

*Corresponding author

Email: w.eilers@reading.ac.uk

Results

Shown below are the original full-length versions of the agarose gel images shown in the manuscript. The red boxes indicate the area of the gels shown in the manuscript figures.

Figure 1b


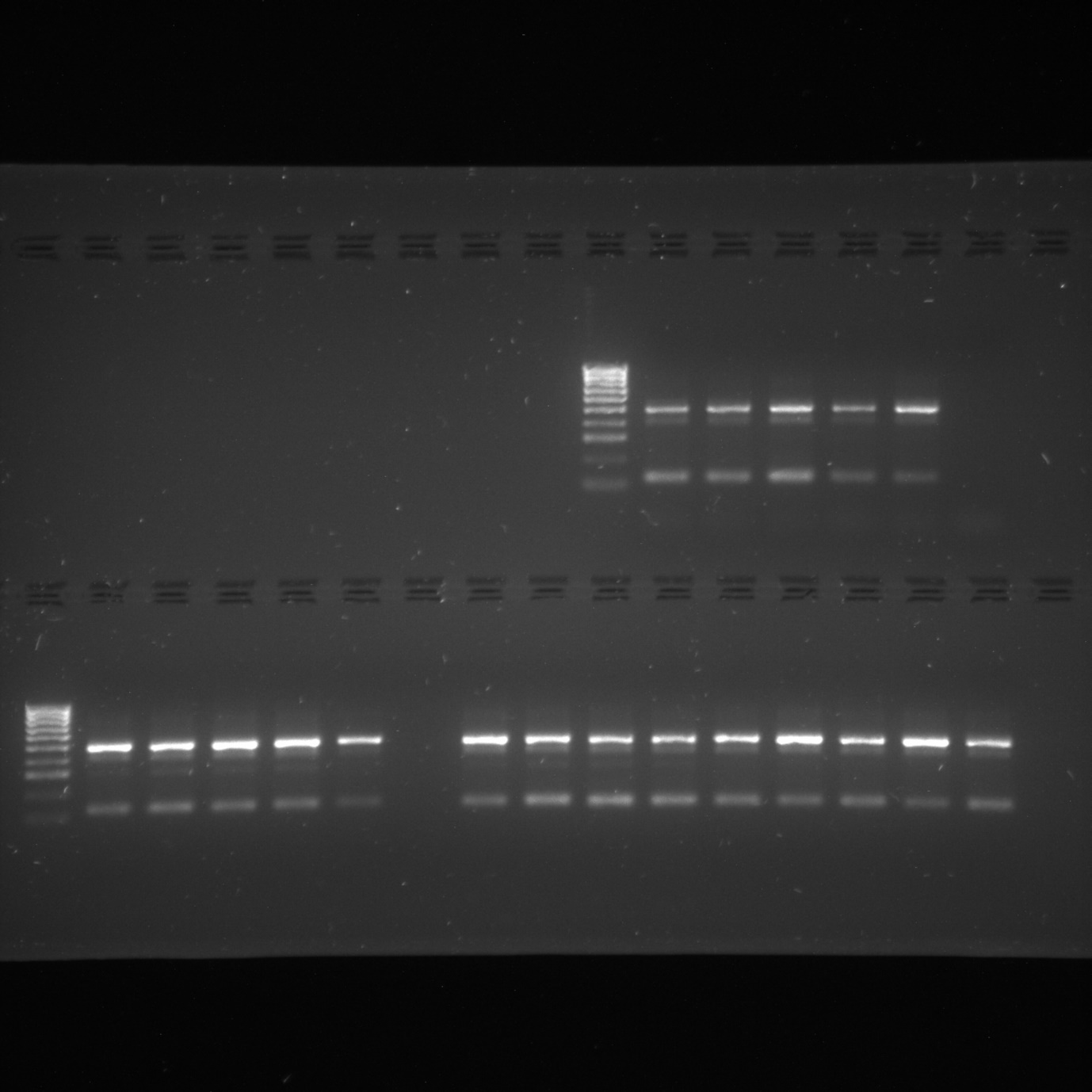


Figure 3e


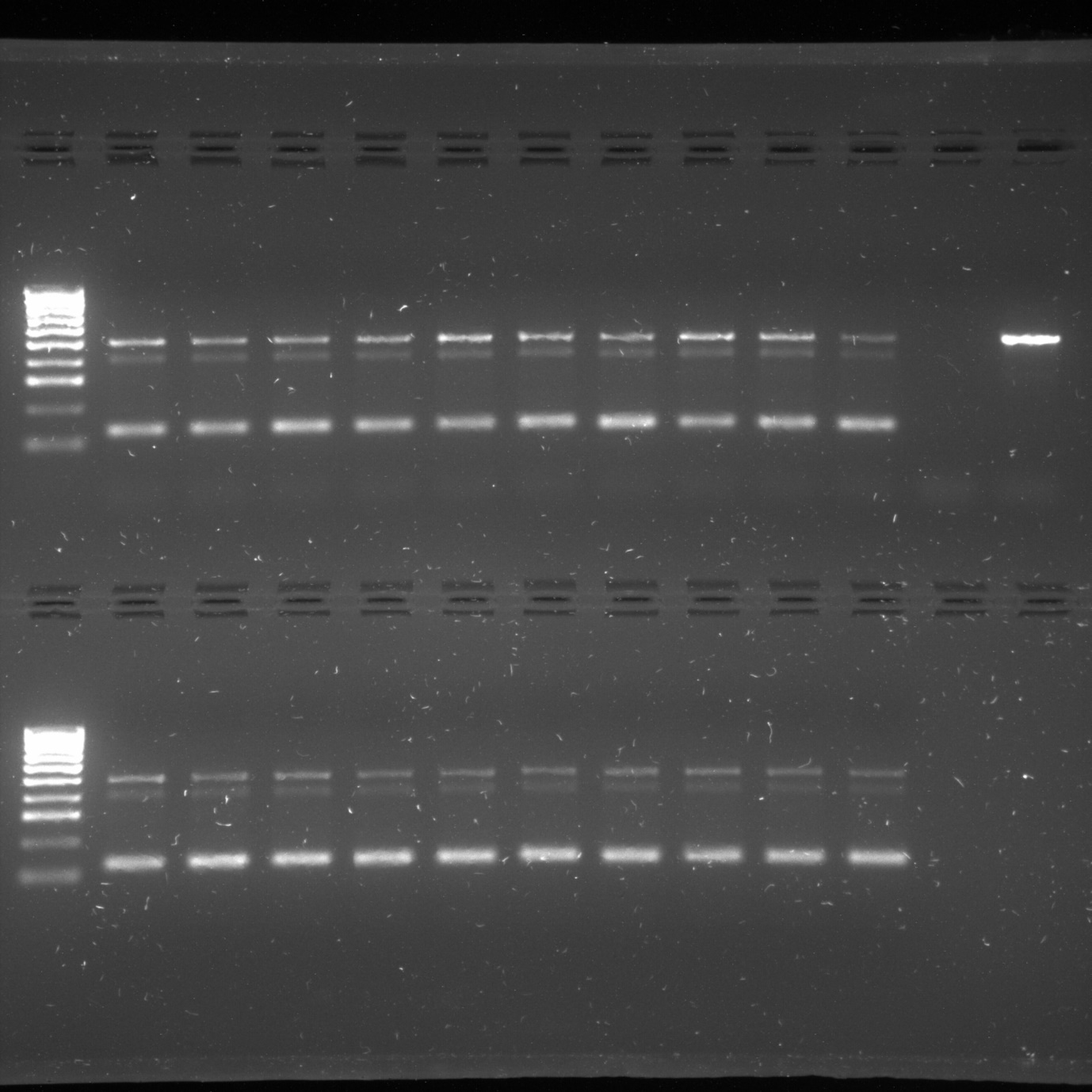


CHOW

HFD

Figure 5i

TA/SOL:


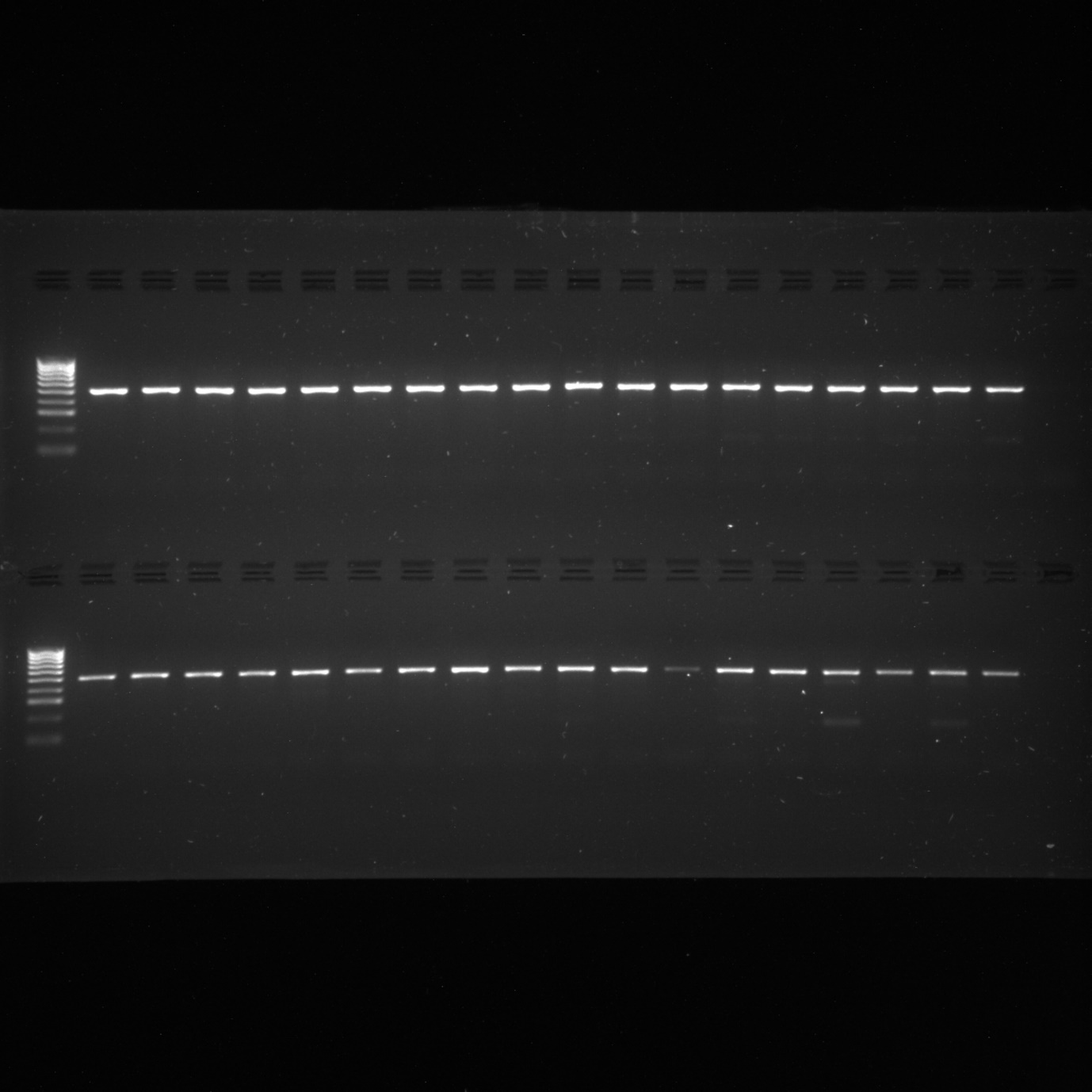


TA

SOL

Epi fat:


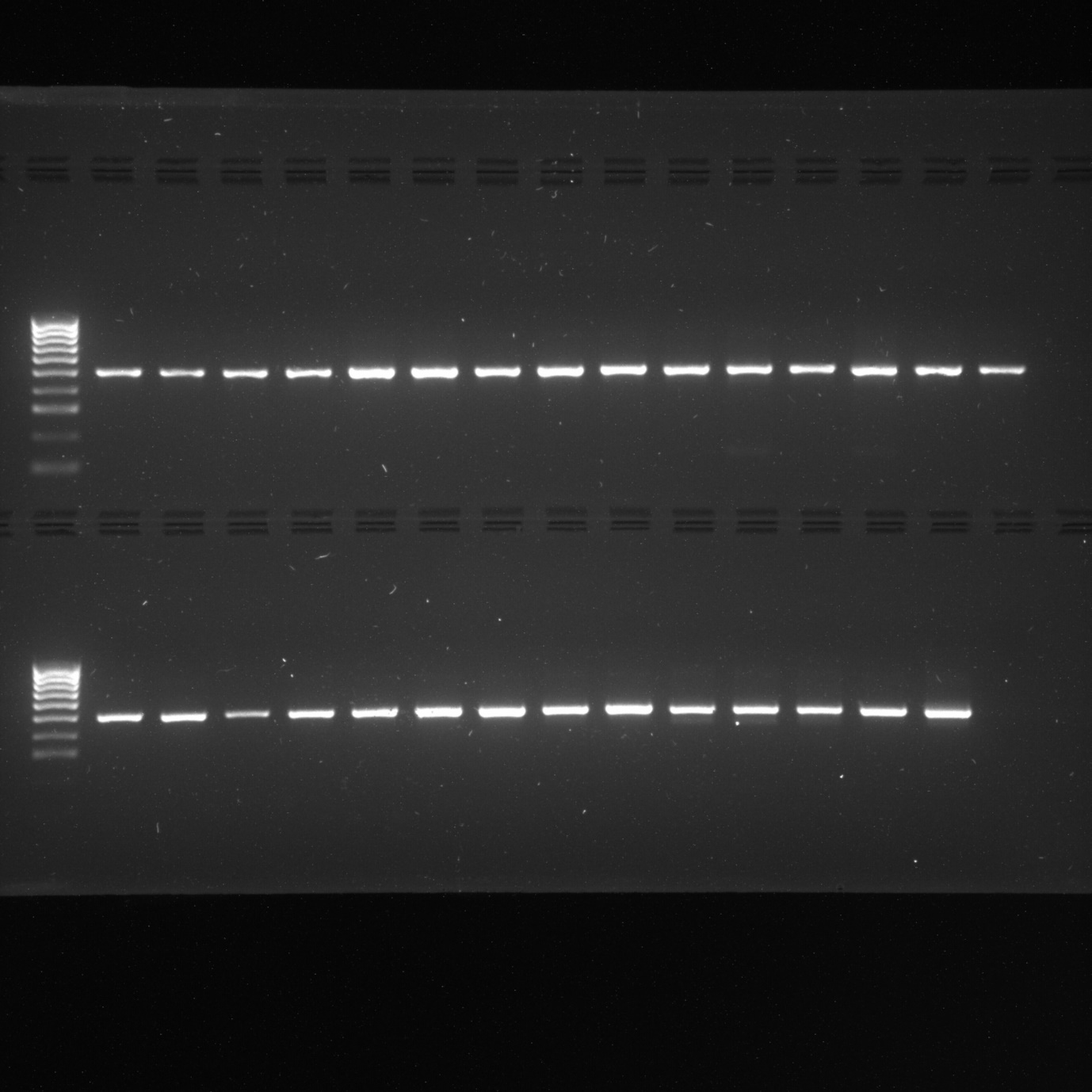


Epi.

Ing fat:


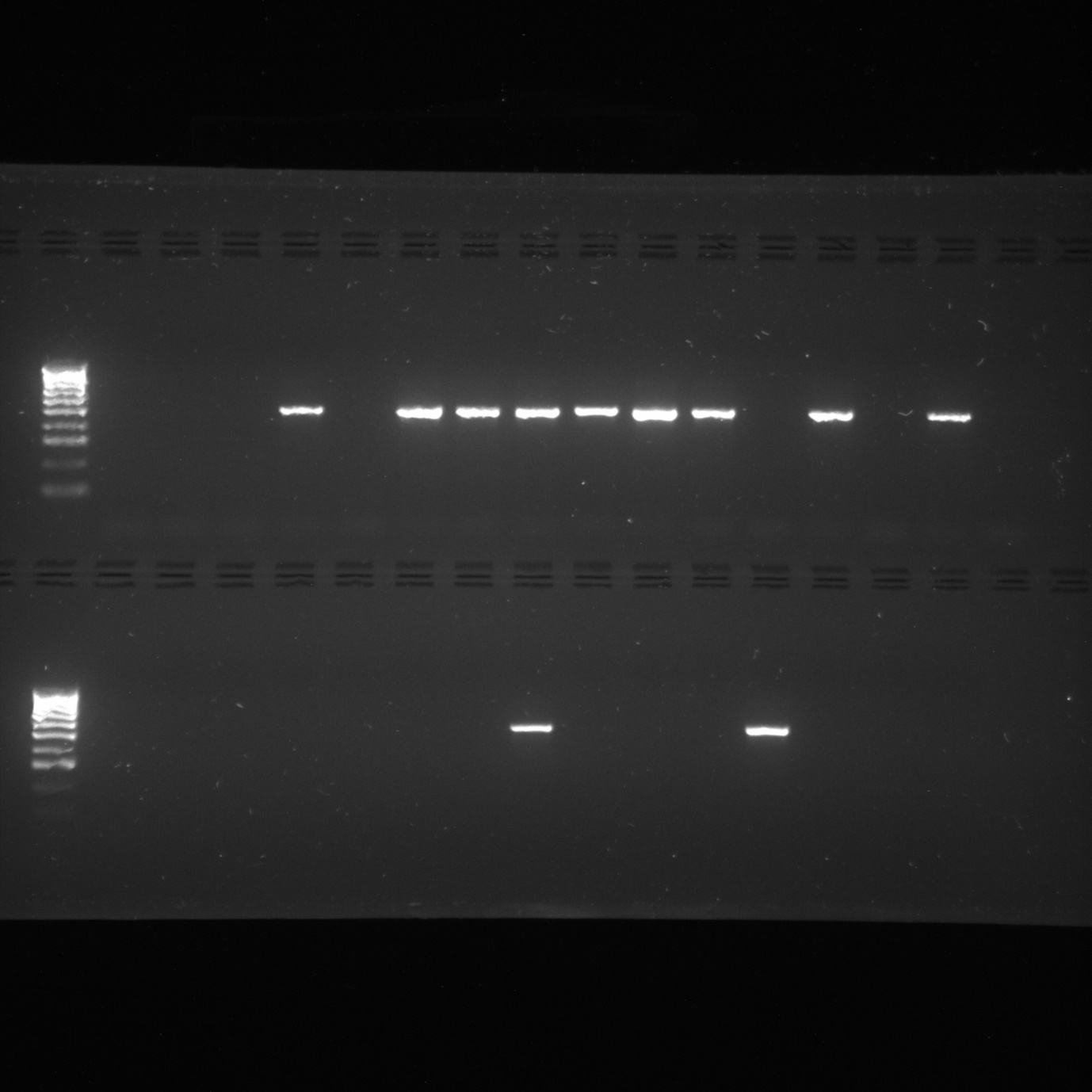


Ing.

Liver:


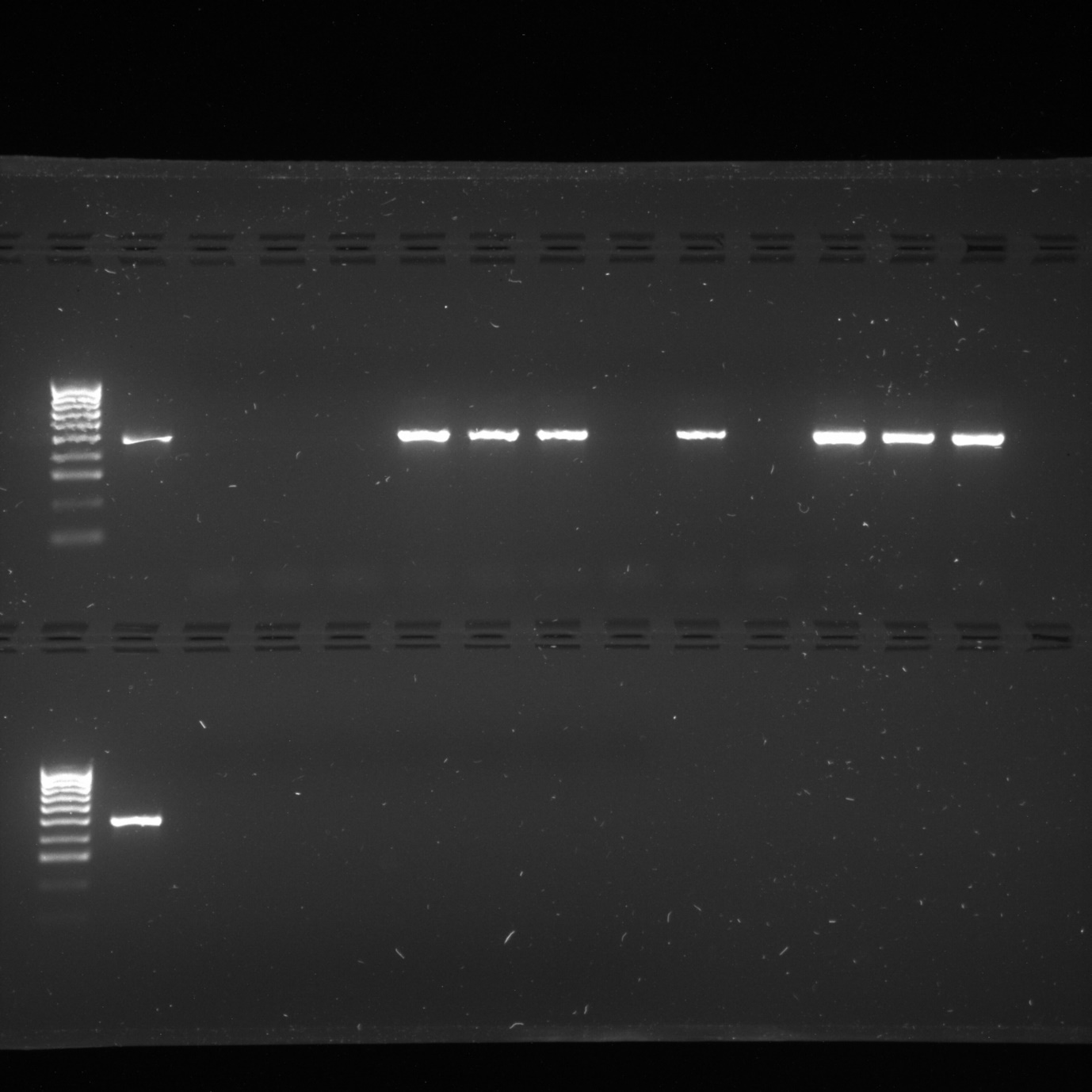


Liver
